# Remote and Long-Term Self-Monitoring of Electroencephalographic and Noninvasive Measurable Variables at Home in Patients With Epilepsy (EEG@HOME): Protocol for an Observational Study

**DOI:** 10.2196/25309

**Published:** 2021-03-19

**Authors:** Andrea Biondi, Petroula Laiou, Elisa Bruno, Pedro F Viana, Martijn Schreuder, William Hart, Ewan Nurse, Deb K Pal, Mark P Richardson

**Affiliations:** 1 Institute of Psychiatry, Psychology & Neuroscience King's College London London United Kingdom; 2 Faculty of Medicine University of Lisbon Hospital de Santa Maria Lisbon Portugal; 3 ANT Neuro UK, Ltd Stevenage United Kingdom; 4 Seer Medical Inc Melbourne Australia; 5 Department of Medicine St. Vincent’s Hospital Melbourne The University of Melbourne Melbourne Australia

**Keywords:** epilepsy, EEG, electroencephalography, brain ictogenicity, wearables, seizure prediction, brain, seizures, mobile technology

## Abstract

**Background:**

Epileptic seizures are spontaneous events that severely affect the lives of patients due to their recurrence and unpredictability. The integration of new wearable and mobile technologies to collect electroencephalographic (EEG) and extracerebral signals in a portable system might be the solution to prospectively identify times of seizure occurrence or propensity. The performances of several seizure detection devices have been assessed by validated studies, and patient perspectives on wearables have been explored to better match their needs. Despite this, there is a major gap in the literature on long-term, real-life acceptability and performance of mobile technology essential to managing chronic disorders such as epilepsy.

**Objective:**

EEG@HOME is an observational, nonrandomized, noninterventional study that aims to develop a new feasible procedure that allows people with epilepsy to independently, continuously, and safely acquire noninvasive variables at home. The data collected will be analyzed to develop a general model to predict periods of increased seizure risk.

**Methods:**

A total of 12 adults with a diagnosis of pharmaco-resistant epilepsy and at least 20 seizures per year will be recruited at King’s College Hospital, London. Participants will be asked to self-apply an easy and portable EEG recording system (ANT Neuro) to record scalp EEG at home twice daily. From each serial EEG recording, brain network ictogenicity (BNI), a new biomarker of the propensity of the brain to develop seizures, will be extracted. A noninvasive wrist-worn device (Fitbit Charge 3; Fitbit Inc) will be used to collect non-EEG biosignals (heart rate, sleep quality index, and steps), and a smartphone app (Seer app; Seer Medical) will be used to collect data related to seizure occurrence, medication taken, sleep quality, stress, and mood. All data will be collected continuously for 6 months. Standardized questionnaires (the Post-Study System Usability Questionnaire and System Usability Scale) will be completed to assess the acceptability and feasibility of the procedure. BNI, continuous wrist-worn sensor biosignals, and electronic survey data will be correlated with seizure occurrence as reported in the diary to investigate their potential values as biomarkers of seizure risk.

**Results:**

The EEG@HOME project received funding from Epilepsy Research UK in 2018 and was approved by the Bromley Research Ethics Committee in March 2020. The first participants were enrolled in October 2020, and we expect to publish the first results by the end of 2022.

**Conclusions:**

With the EEG@HOME study, we aim to take advantage of new advances in remote monitoring technology, including self-applied EEG, to investigate the feasibility of long-term disease self-monitoring. Further, we hope our study will bring new insights into noninvasively collected personalized risk factors of seizure occurrence and seizure propensity that may help to mitigate one of the most difficult aspects of refractory epilepsy: the unpredictability of seizure occurrence.

**International Registered Report Identifier (IRRID):**

PRR1-10.2196/25309

## Introduction

### Background and Rational

Epilepsy is one of the most common neurological disorders, affecting over 65 million people worldwide, and is characterized by recurrent seizures [1]. The unpredictability of these events is one of the key challenges in this disorder. One in 3 people with a diagnosis of epilepsy do not respond fully to antiepileptic drugs and continue to have seizures [2]. Currently, a solution to assess daily seizure risk and understand seizure triggers would be fundamental to improving quality of life and safety for people with epilepsy and allow the testing of new targeted therapies during periods of high seizure risk (chronotherapy) [3] and a better understanding of individual seizure cycles [4,5]. It might prevent accidents and injuries by giving people with epilepsy time to find a safe place before seizure onset [6] and reduce anxiety and stress, which affect 30% to 50% of people with epilepsy [7,8]. Unfortunately, a feasible solution to continuously monitor and assess this risk is not available.

The development of new wearable devices and smartphone apps that could help people with epilepsy to monitor the evolution of their epilepsy would be a clear step in this direction. During the last few years, smartphone apps and wearable devices that allow long-term monitoring of relevant parameters have been developed with the aim to satisfy both the increasingly urgent need for new reliable devices and techniques as well as patient needs and expectations [9].

Smartphone apps now allow people with epilepsy to collect and share with medical professionals information regarding medication, seizure types, and other information that may relate to seizure occurrence [10]. A few studies have used self-reported questionnaires and electronic diaries to explore the association between sleep-wake cycles, menstrual cycles, stress, poor sleep, failure to take treatment, and an elevated seizure risk, revealing a clear relation between some of these variables and seizure occurrence [11-13]. Karoly and colleagues [4] showed that it is possible to identify, using self-reported e-diaries, a subset of patients with robust circadian and multidien seizure cycles. They also reported that estimates of seizure likelihood based on patient-reported cycles were predictive of electrographic seizures [5].

Additionally, some studies have explored the use of wearable devices combining multiple physiological variables to develop automated seizure detection systems [14]. These studies have shown, with different levels of accuracy and sensitivity, that it is possible to detect specific seizure types characterized by stereotyped events (ie, bilateral tonic-clonic seizure) using electrodermal activity, muscle activity, or heart rate [15-18].

Finally, there is strong evidence that factors associated with elevated seizure risk can be objectively detected using electroencephalography (EEG). Video EEG monitoring is the gold standard for the diagnosis of epilepsy. However, conventional video EEG requires patients to stay in the monitoring unit until the collection of multiple seizures is completed. This is an expensive solution and impractical for long-term recording, and it is not well accepted by some patients [19]. Another solution is home video electroencephalographic telemetry. It reduces high costs and long waiting times for hospital admission but presents problems related to long-term electrode attachment on the scalp and correct camera placement while the patient is unsupervised at home, and there are concerns related to regulations regarding data privacy for cloud services [20].

New portable or implantable EEG devices allow for the continuous collection of high-quality data outside of the hospital without direct supervision by a specialist [21,22]. One clinical trial demonstrated a proof-of-principle, real-time seizure prediction system in a cohort of 15 people with epilepsy using an intracranially implanted EEG device (NeuroVista seizure advisory system) [23]. Weisdorf et al [24] and Gangstad et al [25] demonstrated the acceptability and data quality of an implanted subcutaneous EEG system (Uneeg 24/7 EEG SubQ; Uneeg Medical) during a long-term trial in outpatients with focal epilepsy. Askamp and Van Putten [26] also showed that the use of a portable EEG solution for at-home assessment (Mobita 32-Channel Wireless EEG System; Twente Medical Systems International) was well accepted by adult patients with intractable epilepsy as well as neurologists and that data collected were comparable with a normal ambulatory scalp EEG. Finally, an ongoing study called HOMEone aims to provide evidence of the diagnostic and therapeutic yield of a patient-controlled portable EEG device (Fourier One; Nielsen Consumer Neuroscience) with dry electrodes for the purposes of EEG home monitoring of neurological outpatients [27]. All these studies [13,23] have shown that it may be feasible and clinically valuable to monitor patients with epilepsy outside of the hospital or research settings thanks to the development of new portable and easy-to-use devices. Despite this, clear information about the long-term and remote acceptability and performance of mobile technology is still not available in the literature.

To bridge this gap, high data quality and good compliance are needed. The first step is the design of an acceptable and feasible procedure to noninvasively monitor patients at home. Starting from this key point, we decided to design a procedure (EEG@HOME) to investigate whether frequent measurements performed independently by people with epilepsy using a portable EEG cap with dry electrodes (waveguard touch; ANT Neuro), smartphone app (Seer app; Seer Medical), and wrist-worn device (Fitbit Charge 3; Fitbit Inc) in their home environment would be feasible and acceptable.

The successful implementation of an at-home, long-term monitoring procedure like this will enable a novel and innovative approach to epilepsy management. It promises to provide key information to prospectively identify periods of higher seizure risk and improve the management of epilepsy.

### Study Objectives

The main goal of the EEG@HOME study is to develop and test a feasible and acceptable procedure for people with epilepsy to easily undertake twice-daily, at-home EEG coupled with an event app and continuous data collection from a wrist-worn wearable sensor device. All the information gathered about acceptability and feasibility of the procedure will be published and used to refine methods for future and larger controlled studies at home.

The study will produce a substantial amount of continuous data from people with epilepsy over many months. Assuming complete data collection, we estimate having 60 hours of raw EEG data and 4000 hours of raw wrist-worn sensor data collected at home per participant in addition to patient-reported events. Recognizing the uniqueness of the data set, we will curate it and make anonymized data and clinical metadata available to other researchers, with the aim to create an open database for future research.

The unique set of wearable EEG data, sensor data, and self-reported events will be then analyzed to develop a general model to predict periods of increased seizure risk. The association between self-reported events (seizures, medication taking), sensor data (sleep, heart rate), and EEG features will be investigated within and between subjects.

## Methods

### Study Design and Population

EEG@HOME is an observational nonrandomized and noninterventional study. A total of 12 people with epilepsy referred as part of their routine clinical care to the epilepsy clinics at King’s College Hospital and partner hospitals will be enrolled. Patients will be included if they have received a diagnosis of treatment-resistant epilepsy, their age is between 18 and 75 years, and they experience more than 12 seizures (with impaired awareness) per year according to their seizure diary. A current diagnosis of psychogenic nonepileptic attacks (dissociative seizures), inability to comply with the trial procedure or give informed consent, and the use of other electronic medical devices that could interfere with the data collection will result in exclusion from the study.

### Study Overview

Participants will be initially identified among those attending a routine outpatient appointment, hospital video EEG Epilepsy Monitoring Unit admission, or home video EEG monitoring appointment at the participating sites. These individuals will be approached by a member of the on-site research team and given the study participant information sheet. This will be labelled “first approach.”

After the participants have at least 24 hours to read the participant information sheet and consider enrolling, the research team will contact the participants and invite them to attend visit 1 (inclusion and training). All study procedures and eligibility criteria will be discussed, and the EEG device and wrist-worn sensor will be shown to the patients. If the patient is willing to participate, written informed consent to participate in the study will be obtained. Procedures for using the equipment will then be explained. By the end of the inclusion and training visit, the participant should be able to collect their own EEG data, wrist-worn sensor data, and app data independently. An appointment for the next visit will be scheduled after approximately one month. The participant will start to collect their data immediately after visit 1.

After this, the patient will attend monthly follow-up visits up to 6 times (visits 2-7), carried out in the patient’s home or in the research facility of the hospital, according to participant preference. These meetings will allow the research team to confirm the correct collection and download of the event diaries and sensor data. Furthermore, compliance with procedures will be assessed.

**Figure 1:**
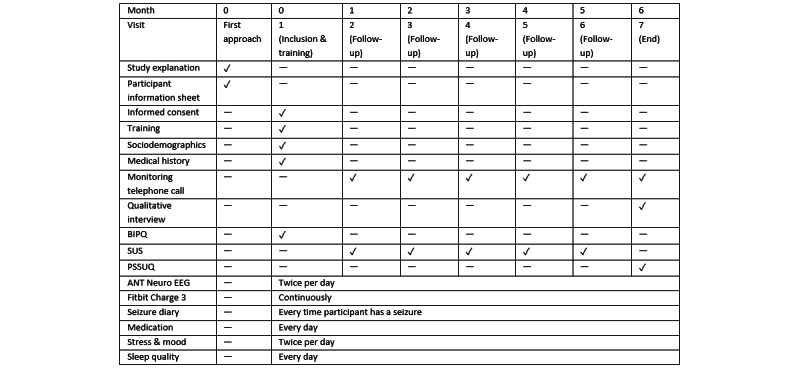
Schedule of events in the EEG@HOME study. BIPQ: Brief Illness Perception Questionnaire; EEG: electroencephalography; PSSUQ: Post-Study System Usability Questionnaire; SUS: System Usability Questionnaires.

At visit 7, the final study visit, acceptability and usability of the technology will be assessed. Participants will be asked to complete a short questionnaire and then a semistructured interview (15 minutes). The detailed flowchart of all the events is presented in Figure 1.

This study will be carried out in accordance with the World Medical Association Declaration of Helsinki (1964); The Research Governance Framework for Health and Social Care (second edition, 2005); the Data Protection Act (2018), which includes the provisions of the General Data Protection Regulation; and the principles of Good Clinical Practice (GCP). All devices are Conformitè Europëenne (CE) marked for the purpose for which they will be used in this study. All investigators will have up-to-date training in GCP. All staff working on the study have received training in study conduct, informed consent, and risk assessment.

### Study Withdrawal

Participants will be free to withdraw from the study at any time. In the case of participant self-withdrawal, a face-to-face appointment will be scheduled to establish the cause of withdrawal, collect qualitative and quantitative data regarding their experience, and collect the EEG system and equipment. All data, including those from study withdrawals, will be included in the final analysis.

### Study Technology

**Figure 2:**
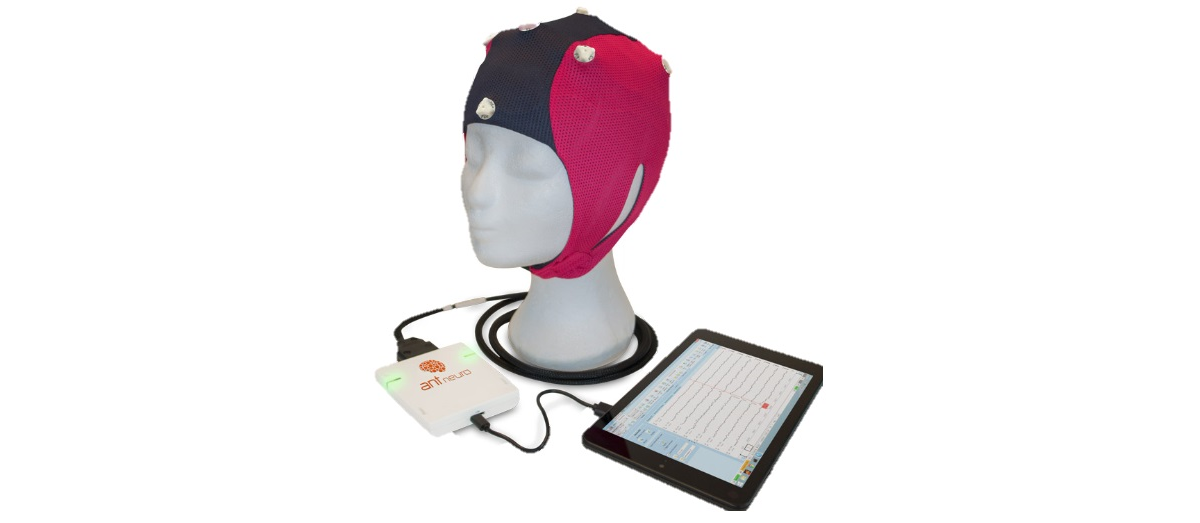
The ANT Neuro eego mini-series and ANT Neuro waveguard touch.

The ANT Neuro eego mini-series (miniaturized EEG recording system) and ANT Neuro waveguard touch (easy-to-use 8-channel dry EEG cap) will be used to record EEG twice daily for 10 minutes [28] (Figure 2). These will provide a quick and easy setup that people with epilepsy can use at home without technical support. The dedicated computer recording software (eego; ANT Neuro) allows the review of the data online and offline and the possibility to parse the data into the standard file format.

The ANT Neuro eego was selected from a shortlist of commercial devices. Following Pinho and colleagues’ work [29], an EEG system used for clinical purposes in outpatients must meet 9 requirements: wireless connectivity, dry electrodes, signal resolution, sampling frequency, comfort, portability, signal artifact attenuation, event detection, and event prediction. Neumann et al [27] also added the necessity of an integrated and structured reporting system and full coverage of the 10-20 System of electrode placement. The ANT Neuro eego meets several of these technical demands required for a clinical outpatient EEG system but not all of them. Technical specifications of the device compared with other available devices are also summarized in Table 1.

**Table 1:** Specifications of commercially available EEG portable solutions for diagnostic or research purposes.

Device (Manufacturer)	Channels	Sample rate	Battery	Resolution	Electrodes	Electrode placement	Weight (g)
Mobita (Twente Medical Systems International)	Up to 32	Up to 2000 Hz	Rechargeable	24-bit	Water-based electrodes	10-20 System	225
Epoc+ (Emotiv)	14	Up to 64 Hz	Rechargeable	16-bit	Saline-based electrodes	10-20 System	170
Fourier One (Nielsed)	19	Up to 500 Hz	Rechargeable	24-bit	Dry silver electrodes	10-20 System	—^a^
Safiro (Compumedics)	32	Up to 512 Hz	Rechargeable	16-bit	Actively shielded electrode wires	10-20 System	270
Enobio (Neuroelectric)	8 to 32	Up to 125 Hz	Rechargeable	24-bit	Handy gel, solid gel, and dry electrode solutions (Ag-AgCl coating)	10-20 System	—
Quick (Cognionics)	8 to 30	Up to 1000 Hz	Rechargeable	24-bit	Dry electrodes (Ag-AgCl coating)	10-20 System	596
Eego amplifier series (ANT Neuro)	8 to 64	Up to 2084 Hz	Connected with a computer or tablet	24-bit	Dry silver electrodes	10-20 System	100

^a^Not available.

A Fitbit Charge 3 will be provided, and during the first approach visit, the need to wear the device continuously will be explained. The Fitbit Charge 3 is a consumer-oriented fitness tracker that provides 24/7 estimates of heart rate, estimates of sleep stages, and activity information (steps, calories, sport activity). The device uses a combination of a microelectronic triaxial accelerometer to capture body motion in 3D space and a photoplethysmogram to extract heart rate. Proprietary algorithms are implemented to identify daily steps taken, sleep, and other activities (eg, running, biking) [30]. This device was selected from a list of wrist-worn devices based on dependability, durability (battery life of up to 7 days), and user acceptability [31]. The Fitbit smartphone app will be installed on the patient’s mobile phone through an anonymized email account.

**Figure 3:**
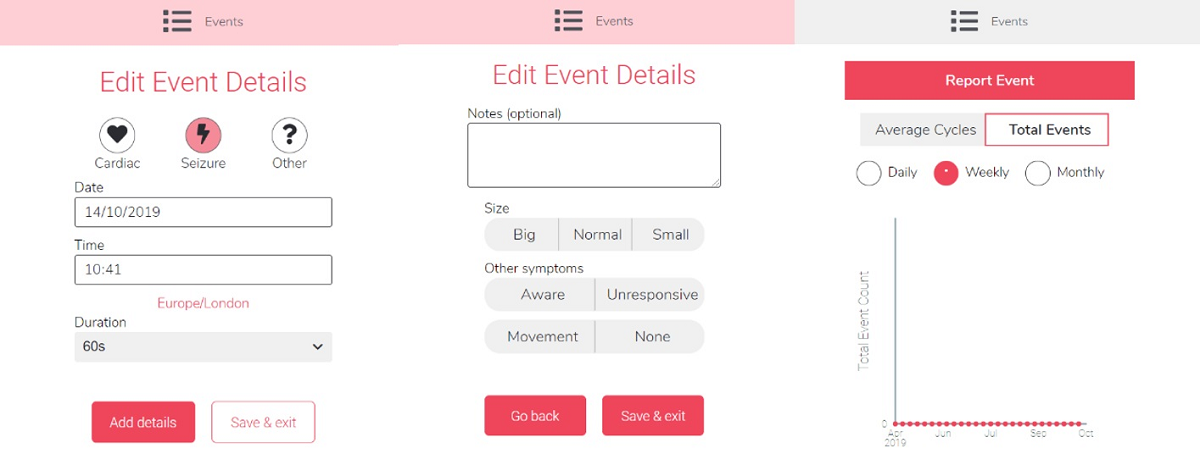
Multiple screens of the Seer app’s events function.

The Seer app (available for Android and iOS devices) [32] is a smartphone app for people with epilepsy to keep track of their seizures and medications. It was created with the aim of helping people with epilepsy manage their symptoms and treatment. It allows patients to report different events, add notes, and receive feedback and visualize it (Figure 3). The Seer app will be downloaded to the patient’s mobile phone using an anonymized email account.

### Data Measured

Electroencephalographic data will be collected using the ANT Neuro eego (approximately 10 minutes of recording while the participant is resting with eyes closed every morning and evening at an interval of approximately 12 hours). First, 1 trained neurologist will visually examine each 10 minutes of EEG recorded, and all the focal interictal epileptiform discharges (IEDs), generalized spike-wave discharges, and focal slowing will be marked. Then, at least one 20-second segment of EEG while the participant is awake with eyes closed that is free of movement and artifacts, signs of drowsiness or sleep, or IEDs will be selected from each recording.

From each segment, different EEG features will be extracted, starting from simple conventional measures to more complex and novel biomarkers. Initially, the power spectra will be calculated. Specifically, our analysis will be focused on 5 frequency bands (1-5 Hz, 6-9 Hz, 10-11 Hz, 12-19 Hz, and 21-70 Hz) defined from previous literature [33,34].

The first measure that will be calculated is the alpha power shift. It is defined as the ratio of average power in the low-alpha power (6-9 Hz) over average power in the high-alpha power (10-11 Hz). Abela and colleagues [35] compared patients with good and poor (4 or more seizures in 12 months) seizure control, showing that alpha power shift is a robust indicator of seizure liability.

In addition, from each 20-second EEG segment, we will infer functional brain networks in which each node corresponds to the brain region underneath each electrode and the edges denote statistical dependencies between the corresponding EEG signals. We will use the phase-locking factor, ignoring all connections at zero lag [36] as well as those that are not statistically significantly different from surrogate data [37]. We will quantify structural properties of the functional brain networks using graph theory metrics, such as the mean and variability of the degree distribution and clustering coefficient [33,38].

Complementary to this graph theory analysis, we will employ a computational modeling framework, the so-called brain network ictogenicity (BNI), which quantifies the propensity of a brain network to generate seizures [39,40]. In this modeling framework, brain models that are able to transit between normal and seizure-like activity are connected using the same connectivity obtained from the functional network that was computed from the EEG signals. This framework is specific to the individual, since it uses the connections obtained from each individual. In order to quantify the contribution of a single brain region (ie, single node in the functional network) to the generation of seizures, we will use the quantity of node ictogenicity (NI), which measures the changes in BNI upon removal of a single node [40]. Therefore, for each 20-second EEG segment, we will obtain 1 BNI value as well as 8 NI values for each node.

In this study, the mentioned EEG features will be evaluated for their predictive value for seizure occurrence and for their potential association with other variables, like treatment, heart rate, sleep, and mood or stress.

Heart rate will be continuously (24/7) estimated using the Fitbit Charge 3 for 6 months. We will specifically use the output of the Fitbit proprietary algorithm, which estimates heart rate from the photoplethysmography sensors approximately every 5 to 15 seconds. Changes in heart rate that could associate with and potentially predict seizure occurrences will be evaluated [41].

Sleep will be also assessed every day. The Fitbit Charge 3 will provide sleep duration (hours and minutes), sleep stages (hours and minutes spent in deep sleep, light sleep, rapid eye movement, and awake), and a Fitbit Quality Sleep Score Index (from 0 [worst] to 100 [best]). Similar to the heart rate analysis, we will investigate changes in sleep that could associate with and potentially predict seizure occurrences.

Through the Seer app, participants will provide a range of patient-reported outcomes with information regarding their mood (twice per day, range of 1-5), stress level (twice per day, range of 1-3), sleep quality (in which participants rate last night’s sleep compared with the previous night, with choices for worse, usual, and better), medication compliance (twice per day, binary yes or no), and seizure events (seizure start, end, and movement) via brief app questionnaires. The questions were selected and adapted to our study from already published studies focused on seizure precipitants and triggers [11,12]. Changes in these patient-reported outcomes that could associate with and potentially predict seizure occurrences will be evaluated.

To provide information about patient characteristics that could determine compliance and feasibility, a set of questionnaires will be administered. Beliefs and attitudes will be assessed using the Brief Illness Perception Questionnaire (BIPQ). It is a validated 9-item scale designed to rapidly assess the patients’ emotional and cognitive illness representations. The questionnaire is structured in 6 sections to assess different factors: consequences (BIPQ 1), timeline (BIPQ 2), personal control (BIPQ 3), treatment control (BIPQ 4), identity (BIPQ 5), concern (BIPQ 6), and emotions (BIPQ 8). Finally, 1 item assesses illness comprehensibility (BIPQ 7) [42].

Two validated questionnaires (System Usability Scale [SUS] and Post-Study System Usability Questionnaire [PSSUQ]) will be used to assess participants’ first impressions after the training and the overall experience of the study, respectively. The SUS is a 10-item usability questionnaire that will be used in this study to evaluate interaction with the ANT Neuro EEG recording system and the Fitbit Charge 3. It is an easy and fast scale to administer and has been validated on small sample sizes with reliable results that allow effective differentiation between usable and unusable systems [43].

The PSSUQ is a short and validated questionnaire that will be used in this study to assess patients’ experiences with the EEG system and the Fitbit Charge 3. It includes 16 items created to measure users’ perceived satisfaction with a system at the end of a study [44]

Finally, a semistructured interview will be scheduled at the end of the study. The researcher will collect direct feedback from the participants about their experience, impression of the devices, comfort, problems and issues, future solutions and improvements, suggestions, reasons for discontinuing wearing a device (if they dropped out), possible concerns, and data privacy and security.

### Data Security and Privacy

Each participant will be assigned a sequential identification code (ie, EEGatHOME00?) used to collect, store, and report participant information. The key to the pseudonymization code will be held in the hospital computer system. Each participant will also receive a nonidentifiable email (ie, eeghome001@xxxx.com) that will be used only for data collection and streaming from the apps to the server. All digital and nondigital information related to study participants will be nonidentifiable in accordance with the General Data Protection Regulation.

Data acquired from the ANT EEG system and Fitbit wrist-worn biosensor will be encrypted, pseudonymized, and uploaded automatically to secure servers managed by the research team and already approved for long-term patient data collection. Data collected from the smartphone app will be also collected and entered in a password-protected database and stored locally and on a secure server. No personally identifying data will be stored or processed within research infrastructure. The research team will assess on a weekly basis the amount of data collected by each participant to evaluate the missing rate and ensure the best data quality.

Clinical information and questionnaires will be collected using pseudonymized forms. Study documents will not contain any identifying information, only study numbers. All questionnaires and documents collected as part of the study process will be stored on password-protected computers.

Although we have access to sufficient infrastructure and storage for the data set for this study, a larger study or real-life rollout of a monitoring system of this kind would require a very large, secure infrastructure that would need to be compatible with legal requirements in multiple countries. These considerations are outside the scope of this study.

### Analysis Plan

At the end of the 6 months, for each participant, the research team expects to have up to 4380 hours of continuous data from the Fitbit Charge 3, 365 EEG segments, 730 daily questionnaires, 7 acceptability questionnaires (BIPQ, SUS, and PSSUQ) and 1 semistructured interview. We also expect that during the study, each participant will have at least 6 seizures, and therefore, the total number of seizures across all patients is expected to be at least 72.

Initially, descriptive statistics will be applied on questionnaires (BIPQ, SUS, and PSSUQ) and demographic information (age, sex, and education) to have an overview of the overall experience. Data missing rate will be also calculated for each device and descriptive analysis will be applied. Using regression analysis, we will investigate if any demographics or other numerical information (sleep, mood, stress) serves as a predictor for participant dropout (if it occurs before the end of the 6 months). We will then estimate for each participant whether there is any relationship between the data missing rate and the self-reported measures (sleep, mood, stress) as well as the monthly acceptability assessment (PSSUQ).

Finally, a thematic analysis will be performed on the recorded semistructured interviews and the weekly call. Audiorecordings will be transcribed, and then a thematic analysis will be performed using NVivo software (version 12; QSR International) by 2 researchers working independently. Initially, the major themes and subthemes will be identified. Once all themes and subthemes are labeled, a secondary analysis will be performed to categorize the dimensions extracted.

The quantitative measures will be computed using data from 3 periods: preictal (within a defined period prior to seizures), postictal (within a defined period following seizures), and interictal (all other times). We will rerun our analysis using 3 different time periods for preictal and postictal periods (24 hours, 72 hours, 7 days); interictal time periods of the same duration as the preictal and postictal periods will be randomly selected from interictal periods approximately equidistant from consecutive seizures. For each peri-ictal period, we will calculate the median of each of the following variables: BNI, variability of NI distribution, mean degree, clustering coefficient, and alpha power shift. We will also calculate the median heart rate, total steps, total sleep hours, mean mood, and mean stress in the 12 hours prior to the EEG measurements.

The primary analysis will be assessed by the area under the receiver operating characteristic curve (AUC) using each of the 5 variables. AUC is the current gold standard for the evaluation of seizure detection and seizure prediction performance.

In an exploratory factor analysis, we will investigate relationships between variables and their independent predictive values using mixed-effects logistic regression models with repeated measurements. For each seizure, the repeated measurements will be the same variables as the primary analysis and the questionnaires (mood, stress, sleep quality, self-assessment of seizure risk, and medication taken) in the preictal, postictal, and interictal periods.

## Results

The EEG@HOME project received funding in 2018 by Epilepsy Research UK (Multimedia Appendix 1) and was approved by the Bromley Research Ethics Committee (REC reference: 19/LO/0554) in March 2020. Pilot data to test the feasibility and practicality of the procedure, from the use of wearable devices to the data analysis, were collected from April to June 2020. The first group of participants were enrolled in October 2020, and the first results are planned to be published by the end of 2022.

## Discussion

### Study Contributions and Implications

EEG@HOME aims to provide an innovative and flexible procedure that, combining the use of new and multiple technologies, will give patients with epilepsy a solution to monitor their condition independently.

Over 72% of the population in the United States has a smartphone [45], and 60% of people with a mobile phone have downloaded a health app [46]. A recent study also supports the acceptability of new solutions for people with epilepsy, reporting that 80% were willing to use a wearable device for seizure tracking and 69% were also willing to use a smartphone app [47]. These studies highlight the fact that clinical populations are ready to use new technology to better manage their condition. Collecting physiological signals in real time by using a wearable device and self-reporting events with an e-diary can improve compliance and accuracy of epilepsy monitoring [48]. Despite this, a reliable procedure to easily monitor seizure occurrence and triggers that clinicians can recommend to people with epilepsy after initial diagnosis is not currently available. Furthermore, the long-term performance of wearable devices for people with epilepsy has been assessed in only a few studies [23,49].

**Figure 4:**
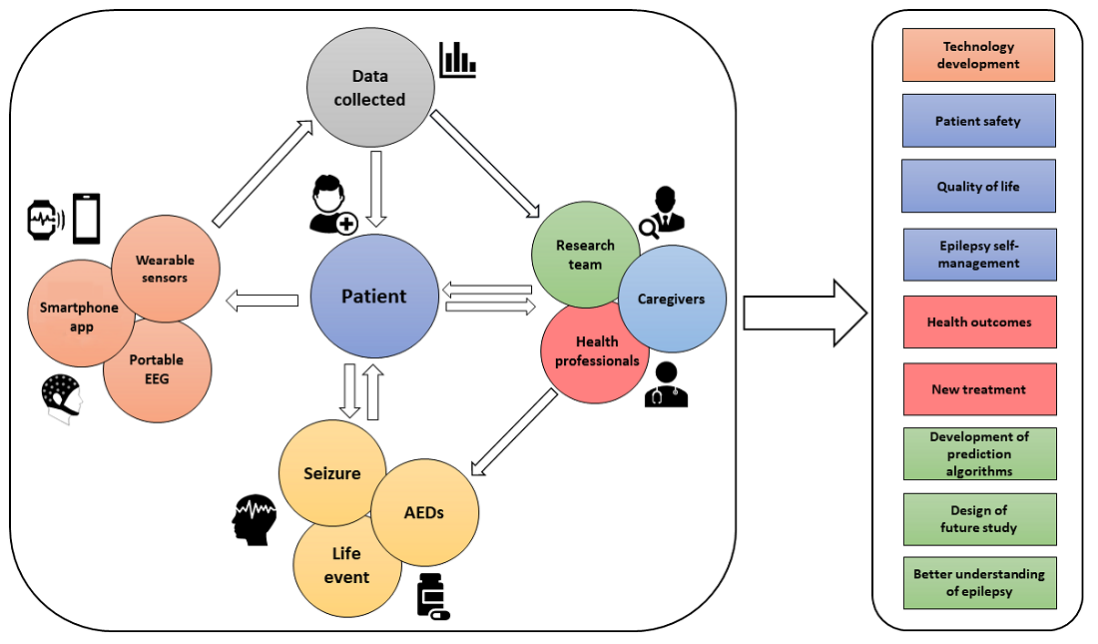
eHealth model of new technology for patients with epilepsy. AED: antiepileptic drug; EEG: electroencephalogram.

To better understand the real impact that these new technologies will have in monitoring people with epilepsy in their daily lives as well as the utility of the data collected, more long-term studies in clinical populations are needed. In Figure 4, we propose a model that outlines the possible outcomes that a prolonged (active and passive) interaction between patients, caregivers, health professionals, and new technology could have in patients’ lives, clinical pathways, and research, as we propose in EEG@HOME.

One of the main innovations in EEG@HOME will be the use of an easy-to-use portable electroencephalogram for long-term intermittent recording. Multiple studies have applied portable EEG solutions [50] to study usability, signal quality, performance, and electrode types, but there is no study providing information about the feasibility and acceptability of portable or wearable solutions during patients’ independent long-term monitoring at home. EEG@HOME will provide for the first time a clear overview of participants’ and developers’ needs to enable “out-of-the-lab” EEG recordings using a portable solution with high compliance and high data quality.

Another key point of this procedure is the combination of multiple technologies to collect detailed data associated with seizure occurrence. Different studies have used self-reported information to study the association between factors such as sleep, mood or stress, and seizure occurrence. The influence of each of these varies between people and is not reliably predictive of seizure risk [51,52]. Other studies have used new wearable devices to collect physiological data associated with seizures. Despite this, a device capable of detecting multiple seizure types with acceptable sensitivity and specificity is currently not available, and the outcomes obtained by research studies are not generalizable to all people with epilepsy [53]. EEG@HOME will combine the use of all these solutions (wearable devices and smartphone app) in combination with continuous biosensor data and a self-reported diary to collect meaningful markers of seizure risk. It will provide a better understanding of the multifactorial nature of seizures, and the association between these data could help in the identification of periods with a higher risk of seizure.

The identification of reliable information about seizure precipitants will also help people with epilepsy to avoid situations that could worsen seizures or cause seizure-related injuries. A continuous monitoring system that could detect seizures and trigger assistance might enhance safety for people with epilepsy [54]. It will increase the self-management and self-efficacy of people with epilepsy. It is well known that poor seizure control is associated with an increased incidence of depression and other mental health disorders [55] as well as an increase in family stress [56] and poor quality of life. It has also been shown that employment rates for people with epilepsy significantly improve to a level comparable to individuals who do not have epilepsy if they achieve good seizure control [57]. Placing people with epilepsy in a more active role in the monitoring and treatment of their condition may increase their quality of life and help them regain a sense of control despite the unpredictability of seizures [58].

EEG@HOME will provide a solution for health care providers and caregivers to easily monitor their patients’ conditions and use the information acquired for better treatment decisions. In clinical practice, clinicians review seizure diaries and clinical history as a proxy for future seizure risk. Decisions about treatment are typically taken after a few months and multiple visits [59,60]. This approach is not the most efficient but is unavoidable in the absence of a validated measure of future seizure risk.

The data set that will be created from the long-term monitoring during the EEG@HOME study will provide more information about the circadian and multiday patterns of seizure occurrence in epilepsy. Karoly and colleagues [5] found that 80% of people with epilepsy had significant and specific temporal cycles in their seizure activity. Most of the cycles were circadian (24 hours) and circaseptan (7 days), but some (approximately 20%) of the seizure cycles were longer than 3 weeks. Increasing the understanding of circadian and long-cycle factors may improve the sensitivity of future seizure prediction algorithms to inform more specific treatment schedules of traditional antiepileptic drugs for individual patients [3].

Finally, multiple factors related to the characteristics of people with epilepsy and device functionality that could affect the experience of people with epilepsy during long-term monitoring will be assessed throughout the study. Having an overview of the main difficulties and critical factors will help in the design of future, larger, long-term studies.

### Conclusion

For these reasons, EEG@HOME is an innovative and flexible procedure that will provide clear information for the design of future data acquisition trials for the at-home management of epilepsy and, potentially, other chronic neurological disorders. The continuous use of wearables and e-diaries will help people with epilepsy manage their condition and provide clinical professionals with reliable information to monitor and control their patients’ therapy. This procedure will improve the interaction between people with epilepsy, caregivers, and heath care providers. All the data collected will also allow the research team to have reliable information for the development of prediction algorithms and the design of future feasibility studies.

## Appendix

Multimedia Appendix 1 Peer Review Report.

## Abbreviations

AUC: area under the receiver operating characteristic curve
BIPQ: Brief Illness Perception Questionnaire
BNI: brain network ictogenicity
EEG: electroencephalography
GCP: Good Clinical Practice
IED: interictal epileptiform discharges
NI: network ictogenicity
PSSUQ: Post-Study System Usability Questionnaire
SUS: System Usability Scale
