# Supplementary material for: Remote and Long-Term Self-Monitoring of Electroencephalographic and Noninvasive Measurable Variables at Home in Patients With Epilepsy (EEG@HOME): Protocol for an Observational Study
Source: JMIR Res Protoc. 2021 Mar 19;10(3):e25309. doi: 10.2196/25309 (PMC8088854; doi:10.2196/25309)
Supplement: Multimedia Appendix 1 [file resprot_v10i3e25309_app1.pdf]

epilepsy research uk

Referee comments on project grant application

Project details

Project code	A1702 Richardson
Project title	Ultra-longterm serial EEG: association of a novel seizure likelihood index with seizure occurrence, stress, sleep and medication

Specific assessment

Importance and relevance: How important is the proposed work and how relevant is it to epilepsy research? *(approx. 50-200 words)*

Seizure prediction represents on of the most challenging and wanted issue in clinical epileptology. Ecologically valid testing a novel biomarker of seizure risk - Brain Network Ictogenicity (BNI) in ultra-longterm serial scalp EEG - is of extremely high importance in epilepsy research.

Originality: Is part or all of the proposed work duplicated by other researchers? *(approx. 50-200 words)*

As far as I am aware this is highly original approach to evaluate current seizure risk and this research can importantly fill a gap in current knowledge of epilepsy with extreme clinical and economical implications.

Plan of investigation: What is your opinion of the quality of the plan of investigation and the likelihood of its successful conclusion within the time specified? Have the appropriate statistical considerations been taken into consideration? Please include specific recommendations for improvement if appropriate. *(approx. 200-400 words)*

The project is carefully structured, time plan is realistic and suggested involvement of 12 subjects with frequent seizures in whom scalp EEG using available ANT system will be recorded at-home twice-daily is very innovative and still accessible. Importantly having objective measurements of seizures, sleep, stress and medication in a parallel with data from 365 ten-minute EEG epochs from each subject will allow authors to analyse data using appropriate statistical methods. It can be expected that results of the study will provide successful and solid conclusions.

**Ethical and R&D approvals (if applicable):** Would you envisage any problems for this proposal with regards to obtaining the necessary approvals? *(approx. 50-100 words)*
No
**Support requested**: Does the level of support requested represent good value for money? (*approx. 50-200 words*)
Project costs are minimized as one PhD student only will be recruited to the project. The compensation to participants and minimal travel costs are necessary and clearly justified and publication costs are relevant. The biggest costs will be necessary for specific easy-to-use out-of-hospital EEG. The authors nevertheless decided to perform a study in three patient subgroups to decrease costs to minimum. As such the level of support requested represents good value for money.

Referee comments on project grant application

Project details

Project code	A1702 Richardson
Project title	Ultra-longterm serial EEG: association of a novel seizure likelihood index with seizure occurrence, stress, sleep and medication

Specific assessment

Importance and relevance: How important is the proposed work and how relevant is it to epilepsy research? *(approx. 50-200 words)*

The problem tackled in this proposal is the monitoring of epileptic patients at home, in order to evaluate if a recently proposed index of seizure "risk", called the Brain Network Ictogenicity (BNI) can identify epochs of time where the probability of seizure occurrence is significantly higher.

By doing so, they intent to address the issue of seizure unpredictability, which is indeed a major issue epilepsy research.

However, according to the literature seizure prediction can hardly be efficient over maximum anticipation time around of 30 minutes, even with intracranial recording, or scalp electrodes using different machine learning methods (see Usmann et al, Comput Math Methods Med. 2017).

Therefore, the topic in itself is relevant to epilepsy research, and the proposed work could have a significant value in this field, knowing that 2 self-recording sessions of 10 min will be limited to predict seizure within a maximum period of 2 relevant hours per day. But the patient's specific association of BNI with external parameters (stress, sleep and medication) through long-term recording is very interesting.

Originality: Is part or all of the proposed work duplicated by other researchers? *(approx. 50-200 words)*

Seizure prediction, in some form, is a topic that has been extensively investigated over the last 20 years. After many studies providing mixed results, in the last few years a few papers (mainly with invasive recordings) have provided evidence that it might be possible to identify epochs where seizures would be more prone to occur. The idea of a device warning patients of increased seizure risk is also not new.

However, the idea of using a non-invasive device (EEG cap) at home to identify such epochs based on 6-months follow-ups is an interesting idea, since such long-term monitoring is not possible in the clinic, and can provide informations with co-occurent events (stress, sleep...).

Therefore, the authors propose a relatively original idea with respect to the state-of-the art, while focusing on a question of significant clinical interest.

Plan of investigation: What is your opinion of the quality of the plan of investigation and the likelihood of its successful conclusion within the time specified? Have the appropriate statistical considerations been taken into consideration? Please include specific recommendations for improvement if appropriate. *(approx. 200-400 words)*

A 3-year study in the context of a PhD thesis seems comfortable to accomplish the presented work, since the number of patients to be recruited and number of datasets to analyze (n=12) are relatively low (see below). The method to compute the BNI has already been published; therefore the risk of delays in data analysis is minimal.

Theta-band functional connectivity based on 9-electrodes scalp EEG recordings might be affected in some of the subtypes of epilepsy, but not for all forms of the disease. Therefore this seizure-prediction method might not account for all epilepsy forms. In the project, patient selection is not mentioned. Before a long-term follow-up of patients, it might be important to define precisely the criteria of selection, in order to improve the chance of successful BNI seizure prediction.

The biggest challenge is instead subject recruitment (which requires to identify motivated individuals) and obtaining 12 usable datasets in a reasonable time frame to proceed with the project, which according to the Gantt diagram should be achievable. It should be noted that the applicant have a solid publication and funding track record, and that there is no doubt on their capability to successfully and efficiently supervise the student who will be recruited on this project.

A sample size calculation has been provided, which is clearly and well presented. However, it might be a good idea to increase the planned number of patients (n=12 so far, which is relatively low) to be recruited, since no potential dropout is planned, either in terms of logistics or in terms of budget. We would recommend accounting for the possibility of dropout both in terms of recruitment and financially. Due to the long-term follow-up of patients (6 months), there is an increased risk of decreased compliance over time, or even dropout.

Ethical and R&D approvals (if applicable): Would you envisage any problems for this proposal with regards to obtaining the necessary approvals? *(approx. 50-100 words)*

We are not anticipating any issues in terms of ethics approval, since the procedures involved in the long-term follow-up are completely non-invasive. Therefore, this should not induce any delays regarding project execution.

Support requested: Does the level of support requested represent good value for money? (*approx. 50-200 words*)

Given the potential of the method that could result from conclusive (if it is the case of course) results in this study, the level of support requested is reasonable, especially since it will contribute to support the entire training of a PhD. Therefore, the applicants present a reasonable budget with significant clinical potential and HQP training.

epilepsy research uk

Professor Mark Richardson
Division of Neuroscience
King's College London
Maurice Wohl Clinical Neurosciences Institute
5 Cutcombe Road
London
SE5 9RX

10 April 2018

Dear Mark,

Our Scientific Advisory Committee (SAC) met on Friday 23 March 2018, and I am delighted to inform you that you have been awarded **£149,372**, over **36 months**, to carry out your proposed study entitled 'Ultra-longterm serial EEG: association of a novel seizure likelihood index with seizure occurrence, stress, sleep and medication'.

According to your application your anticipated start date is 1st February 2019. However, please inform me if there is likely to be any change to this date and inform me of the exact start date. Please note that no grant monies can be allocated until I have received your fully-signed terms and conditions (which accompany this letter), and a copy of the relevant licences/approvals (including ethics and NHS governance if applicable) needed for this study.

With regards to the terms and conditions of funding, please liaise with Kasia Haremza (the finance contact you provided in your full application – kasia.haremza@kcl.ac.uk) and send **one signed copy** to me, Caoimhe Bennett, at Epilepsy Research UK, Chiswick Town Hall, Heathfield Terrace, London, W4 4JN. Either I or our CEO will then countersign the copy and send it back to you at the address you provide.

Please note that once your grant has commenced, invoices should be raised by your institution **quarterly in arrears**. I have also informed Kasia of this.

On behalf of Epilepsy Research UK I would like to congratulate you on your success, and I very much look forward to working with you.

With very best wishes,

Caoimhe Bennett
Research Manager
Epilepsy Research UK
